# Microarray and Bioinformatics Analysis of Circular RNA Differential Expression in Newborns With Acute Respiratory Distress Syndrome

**DOI:** 10.3389/fped.2021.728462

**Published:** 2021-11-02

**Authors:** Huan Zhou, Bwalya Chanda, Yu-fei Chen, Xue-juan Wang, Ming-yu You, Yi-han Zhang, Rui Cheng, Yang Yang, Xiao-qing Chen

**Affiliations:** ^1^Department of Pediatrics, The First Affiliated Hospital of Nanjing Medical University, Nanjing, China; ^2^Department of Neonatology, Children's Hospital of Nanjing Medical University, Nanjing, China

**Keywords:** circRNA, neonatal acute respiratory distress syndrome, bioinformatics analysis, lung injury, neonate

## Abstract

Previous studies pointed out that a variety of microRNAs (miRNAs) are involved in the pathogenesis of neonatal acute respiratory distress syndrome (NARDS) and play different roles in the pathological process. However, there have been few studies reporting the connection between circular RNA (circRNA) and NARDS, so the expression profile of circRNAs in newborns with acute respiratory distress syndrome remains largely unknown. In the present study, 10 samples obtained from remaining clinical blood samples of newborns hospitalized in a neonatal ward of the First Affiliated Hospital of Nanjing Medical University from January 2020 to October 2020 were divided into the “NARDS” group and “non-NARDS” group according to the Montelux standard and then were analyzed in microarray, and 10 other samples collected from the same place and from January 1, 2021 to August 31, 2021, were used to do RT-qPCR experiment. circRNA expression profiles, in which 741 circRNAs were downregulated and 588 were upregulated, were screened with circRNA high-throughput sequencing. Subsequently, Gene Ontology and Kyoto Encyclopedia of Genes and Genomes analysis of parent genes of the differentially expressed circRNAs revealed that these circRNAs may be related to the process of protein synthesis and metabolism in NARDS. Moreover, five circRNAs—hsa_circ_0058495, hsa_circ_0000367, hsa_circ_0005389, hsa_circ_0059571, and hsa_circ_0006608—were selected randomly among the top 10 circRNAs of the downregulated or upregulated expression profiles. Then, bioinformatics tools were used to predict correlative miRNA and its target genes, which were also subjected to the same bioinformatics analysis for further study. The top 30 enriched KEGG pathway analyses of the 125 target genes suggested that these target genes are widely involved in the synthesis and secretion of endocrine hormones, and the top 30 enriched GO terms based on the 125 target genes are also focused on the protein and DNA processing. Thus, the present results show that circRNAs could promote the inflammation of NARDS which may provide a new therapeutic direction and it can be used as molecular markers for early diagnosis of NARDS, but further molecular biology verification is needed to define the specific role of differentially expressed circRNAs in NARDS.

## Introduction

Acute respiratory distress syndrome (ARDS) is a severe respiratory disease threatening life characterized by diffuse alveolar injury and immune cell infiltration. In other words, it has a pathological feature that increased microvascular permeability caused by inflammation and exudation of protein-rich fluid in alveoli, resulting in intractable hypoxemia. The disease could be induced by a variety of factors. According to the Berlin Definition, ARDS is classified as stages of mild, moderate, and severe, which are associated with the ratio of arterial partial pressure of oxygen to fraction of inspired oxygen (PaO_2_/FiO_2_) (1). In addition, a previous study found that the incidence of ARDS exceeds 10% of all ICU admissions, accounting for nearly 25% of all patients with mechanical ventilation (2). Simultaneously, it is about 40% that the mortality rate of ARDS patients during hospitalization increased with the severity of ARDS. Furthermore, there is no age limit for ARDS, and it can occur in the neonatal period. Neonatal acute respiratory distress syndrome (NARDS) was defined for the first time in 1989, which opens the door to further study (3). The characteristics of NARDS are more severe clinical symptoms, a longer length of hospital stay, and higher mortality compared to children or adults with ARDS (4). Recently, the Montelux standard established in 2017 has redefined NARDS and distinguished it from neonatal respiratory distress syndrome (NRDS) and tachypnoea of the neonate (TTN), which provides the basis for early diagnosis (5–7). However, NARDS is not a single disease but a clinical syndrome accompanied by systemic inflammatory response syndrome, which increases the frustration of early diagnosis. For the management of NARDS, there is still no specific treatment for this disease (4). What is more, NARDS has a complicated process, which is accompanied by diffuse alveolar damage (DAD) and systemic inflammatory response syndrome in the lungs. It can cause or aggravate the injury and inflammation of lung epithelium and vascular endothelium (8, 9), which forms a harmful round, so early diagnosis and on-time treatment of NARDS are important.

Circular RNA (circRNA) is a kind of RNA with a special closed-loop structure in which the downstream splice donor site and upstream splice acceptor site are covalently linked, which results in high stability. This is because the back-splicing closed-ring structure protects these molecules from exonuclease-mediated degradation (10). CircRNAs have three sequences: a single exon or multiple exons, exon–introns, and introns. The last two sequences residing in the nucleus can promote the transcription of their parental genes (11). In non-coding RNAs and the majority of circRNAs, single-exon or multiple-exon circRNAs have several functions, but the main effect of circRNA is to adsorb microRNAs (miRNAs) to exert their biological function and participate in transcriptional regulation as a sponge (12, 13). Existing studies have demonstrated that a variety of miRNAs are involved in the pathogenesis of NARDS and play different roles in its pathological process (14). However, no studies have shown that circRNA is involved in the pathogenesis of NARDS. In this study, it was applied to clarify the connection between circRNA and NARDS that circRNA high-throughput sequencing and bioinformatics analysis, containing Gene ontology (GO), Kyoto Encyclopedia of Genes and Genomes (KEGG) analysis and so on.

## Materials and Methods

### Patient Sample Collection

**Figure 1:**
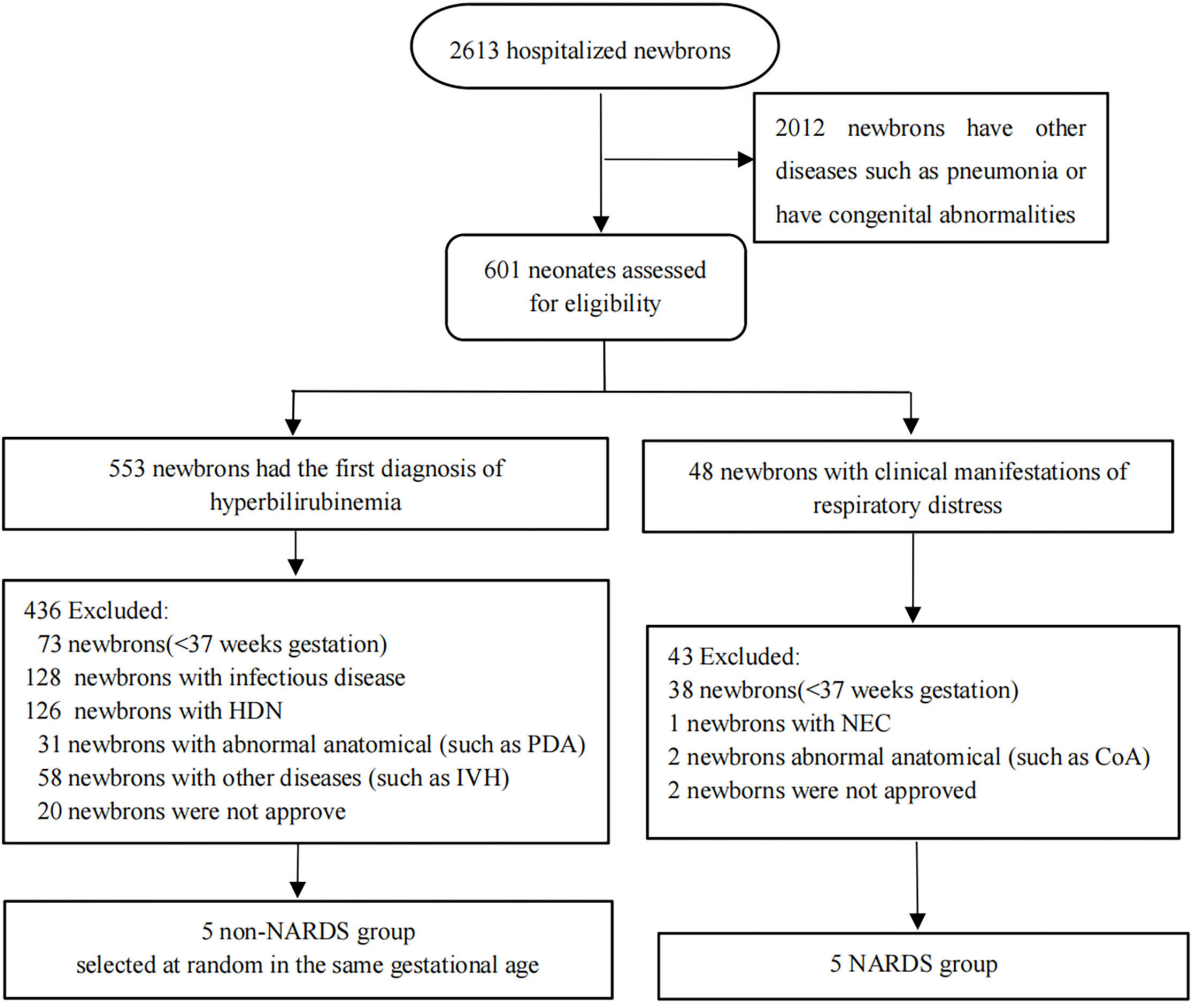
Flow diagram of the patient sample collection applied in the study. In the NARDS group, five newborns were included, and five babies with the same gestational age and only hyperbilirubinemia were selected at random to form a non-NARDS group. HDN, hemolytic disease of newborn; IVH, ventricular hemorrhage; PDA, patent ductus arteriosus; NEC, necrotizing enterocolitis; CoA, coarctation of aorta; NARDS, Neonatal Acute Respiratory Distress Syndrome.

All samples were obtained from patients hospitalized in neonatal intensive care units (NICUs) of the First Affiliated Hospital of Nanjing Medical University from January 2020 to October 2020. A total of 10 blood samples were taken on the day of diagnosis from the remaining clinical blood samples of 10 newborns, and these samples were divided into “NARDS” group and “non-NARDS” group according to the Montelux standard (6). Then, the five pairs of blood samples were analyzed in microarray. In the NARDS group, patients were included according to the following criteria: newborns who have ARDS diagnosed through the Montelux standard and gestational age > 37 weeks (15). Patients with other severe diseases or abnormal anatomical diseases, such as tetralogy of Fallot and coarctation of the aorta, as well as premature infants were excluded. In the control group, we randomly selected five newborns among those who have the same gestational age but with only hyperbilirubinemia. The workflow is shown in Figure 1. By the way, all blood samples have been frozen in the −80°C refrigerator following a specific process which includes centrifugation at 3,000 × g for 10 min at 4°C and then separation of clear upper liquid into an RNase-free tube. In addition, five pairs of blood samples in the same place were used to run RT-qPCR, which were collected using the same method from January 1, 2021 to August 31, 2021. The study was approved by the Clinical Research Ethics Committee of the First Affiliated Hospital of Nanjing Medical University (2021-SR-267).

### CircRNA Microarray

Total RNA was extracted from 250-μl plasma samples using TRIzol® Reagent (Invitrogen; Thermo Fisher Scientific, Inc., Waltham, MA, USA) based on the manufacturer's protocol and quantified using NanoDrop ND 1000. The sample preparation and microarray hybridization were performed based on the standard protocols of Arraystar. Briefly, total RNA was digested with RNase R (Epicenter, Inc., Madison, WI, USA) to remove linear RNAs and enrich circular RNAs. Then, the enriched circular RNAs were amplified and transcribed into fluorescent circRNAs using a random priming method (Arraystar Super RNA Labeling Kit; Arraystar, Rockville, MD, USA). The labeled circRNAs were hybridized onto the Arraystar Human circRNA Array V2 (8 × 15K, Arraystar). After having washed the slides, the arrays were scanned by the Agilent Scanner G2505C. Agilent Feature Extraction software (version 11.0.1.1) was used to analyze acquired array images. Quantile normalization and subsequent data processing were performed using the R software limma package. Differentially expressed circRNAs between two samples were identified through fold-change filtering. Then, a box plot was quickly used to visualize the distributions of the intensities from the two samples. Hierarchical clustering was performed to show the distinguishable circRNA expression pattern between two samples. Differentially expressed circRNAs with statistical significance between two groups were identified through scatter plot filtering. In this study, the criteria for screening the differential expression of circRNAs between two groups were defined as absolute fold change >2 and *p* < 0.05 (16). By the way, this part was implemented by the company named KangChen Bio-tech in Shanghai, China.

### Bioinformatics Analysis

To identify the functional categories of differentially expressed circRNAs, Gene Ontology (GO; http://www.geneontology.org/) and Kyoto Encyclopedia of Genes and Genomes (KEGG; http://www.genome.jp/) were used. In addition, five circRNAs were selected randomly among the top 10 circRNAs ranked by “fold change” of the downregulated or upregulated expression profiles. At the same time, they are also exonic circRNAs, which are the top five circRNAs of the same type in the expression profiles. TargetScan (http://www.targetscan.org) and RNAhybrid (http://bibiserv.techfak.) were used to predict five circRNAs–correlative miRNAs selected from the top five of the co-result. At the same time, the five target genes of correlative miRNAs in the circRNA–miRNA–mRNA network drawn using Cytoscape software (version 3.8.2) were forecasted by TargetScan and miRDB (http://mirdb.org/). Then, these target genes from the top five of each intersection also were subjected to GO analysis and KEGG analysis.

### RT-qPCR

The extraction and quantification of total RNA were processed as previously described. Later, 1 μg total RNA was converted into cDNA using the HiScript® II Q Select RT SuperMix for qPCR (R232-01; Vazyme, Nanjing, China) according to the manufacturer's protocol−4 μl 5 × HiScript II Select qRT SuperMix, 1 μl random hexamers (50 ng/μl), and 1 μg RNA, which were run at 37°C for 15 min and 85°C for 2 min. Subsequently, the RT-qPCR reaction was performed using AceQ® qPCR (Q131-01; Vazyme) in a protocol that is to add 1 μl cDNA to 4 μl master mix, including 2.5 μl SYBR® Green Master Mix (Low ROX Premixed; Q131-01; Vazyme), 0.2 μl reverse and forward primers, and 1.1 μl diethylpyrocarbonate water. Then, qPCR was performed under the conditions which included an initial step at 95°C for 5 min, 40 cycles at 95°C for 10 s, and at 60°C for 30 s, and final extension at 95°C for 15 s, 60°C for 1 min, and 95°C for 15 s, with a LightCycler 480 II (Roche). The primer sequences designed through Primer Premier 6 are listed in Table 1. GAPDH is used as the internal control. So far, 10 blood samples from newborns were used to perform RT-qPCR validation.

**Table 1:** Primers for RT-qPCR.

**circRNA ID**	**Primers**
hsa_circ_0005389	F: AGTCCATTCCACGCCTAC
	R: GAGCACGATCACGAACAT
hsa_circ_0000367	F: CTTGTCCAGGTCCATTCC
	R: CCCTTTCAGAGTCATATTGC
hsa_circ_0059571	F: TCTGCTGCTGTGTTATGG
	R: TGGTAGGAGTGGAGTGATT
hsa_circ_0006608	F: ACTATCACCGACCGTTCT
	R: CACATTGTTGGACACAGTAG
hsa_circ_0058495	F: CATTACAAGCCAGCCTCT
	R: GGTAACACTTCTCCACACTA
GAPDH	F: GAACGGGAAGCTCACTGG
	R: GCCTGCTTCACCACCTTCT

*F, forward; R, reserve*.

### Statistical Analysis

In the present study, when the clinical characteristics of the patients were statistically analyzed, all quantitative data were presented as the median and interquartile range (IQR). Moreover, the data were analyzed using SPSS 17.0 (SPSS, Inc., Chicago, IL, USA) and GraphPad Prism version 8.0 software. A two-tailed Student's *t*-test was applied to analyze differences in circRNA expression levels between the two groups. *p* < 0.05 was considered as a statistically significant difference.

## Results

### Patient Characteristics

A total of 10 patients were included in the microarray in which five newborns were diagnosed with NARDS and five newborns had hyperbilirubinemia. Their clinical and physiological characteristics are outlined in Table 2. In the NARDS group, the five newborns were all complicated with pneumonia, in which the ratio of male to female was 4:1, the median of admission age was over 1 day but not more than 3 days, and two cases suffered from persistent pulmonary hypertension, which reflected the risk factors of NARDS.

**Table 2:** Clinical characteristics of 10 patients in the present study.

	**“NARDS”**	**“Non-NARDS”**
	**group**	**group**
Gestational age at delivery [weeks (median, IQR)]	38.5[37.6;39.2]	38.5[37.6;39.2]
Sex (female:male)	1:4	4:1
Birth weights [grams (median, IQR)]	3540[3225;4100]	2985[2578;3246]
Admission age [days(median, IQR)]	1.2[0.6;2.1]	6.6[5.4;7.6]
Delivery (vaginal:cesarean section)	1:4	4:1
Apgar score	>8	>8
**Maternal diseases**		
Gestational diabetes mellitus (%, *N*/i)	40(2/5)	40(2/5)
Hyperlipidemia (%, *n*/*N*)	20(1/5)	0(0/5)
**Concomitant diseases**		
Neonatal pneumonia (%, *n*/*N*)	100(5/5)	0(0/5)
Neonatal pneumothorax (%, *n*/*N*)	40(2/5)	0(0/5)
Persistent pulmonary hypertension in neonates (%, *n*/*N*)	40(2/5)	0(0/5)

*IQR, interquartile range*.

### Identification of Differentially Expressed CircRNAs

**Figure 2:**
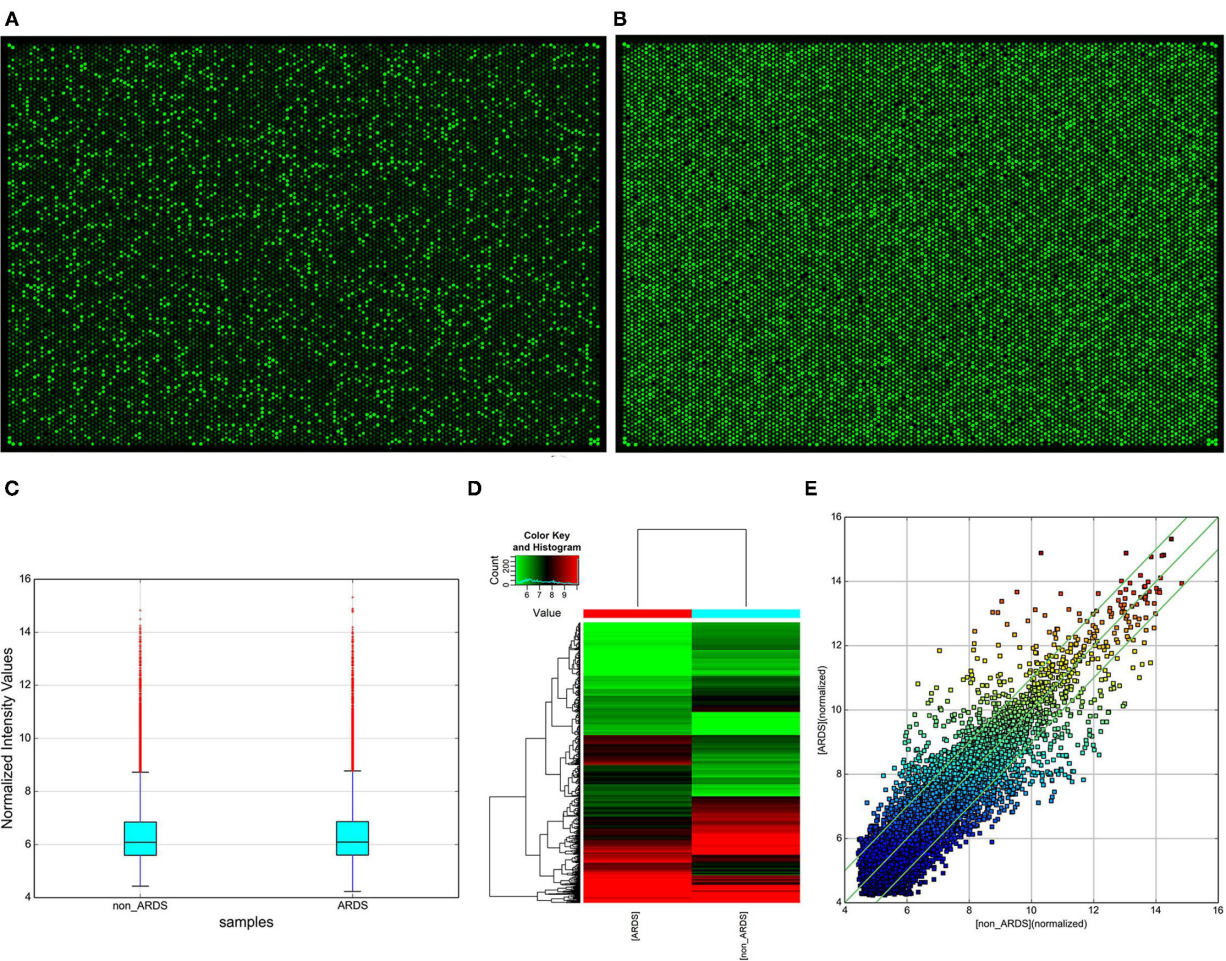
Identification of differentially expressed circRNAs. **(A)** High-throughput human circRNA microarray-array image of NARDS. **(B)** High-throughput human circRNA microarray-array image of non-NARDS. **(C)** Box plot, a method of describing data in terms of minimum, first quartile (25%), median (50%), third quartile (75%), and maximum. The abscissa is the sample name, and the ordinate is the value of the signal value of the probe after log2. The upper and lower sides of the rectangular box correspond to the upper and lower quartiles of the data (Q1 and Q3). The median line inside the rectangular box is the median, the upper line is the Q1 + 1.5 interquantile range (IQR), and the underline is Q3–1.5 IQR, where IQR is the middle quartile range, IQR = Q3–Q1. **(D)** Hierarchical clustering analysis arranges samples into groups based on their expression levels, which allows us to hypothesize about the relationships among samples. Hierarchical clustering was performed based on all circRNAs from two different samples. The result of hierarchical clustering shows a distinguishable circRNA expression profiling between samples. **(E)** Scatter plot, the values of the X and Y axes in the scatter plot are the normalized signal values of the samples (log2 scaled) or the averaged normalized signal values of groups of samples (log2 scaled). The green lines are fold change lines. The circRNAs above the top green line and below the bottom green line indicated more than 2.0-fold change of circRNAs between the two compared samples, circRNAs, circular RNAs.

When comparing differences in expression between the groups for each circRNA, they were computed by *t*-test showing that the “fold change” in high-throughput human circRNA microarray has a statistical significance (Figures 2A,B). Subsequently, a box plot is used for visualizing the intensities of expression values from the samples after normalization, which showed a similar distribution (Figure 2C). In addition, hierarchical clustering (Figure 2D) and scatter plot (Figure 2E) were applied. Moreover, the results exhibited that two circRNA expression profiles are different. To sum up, the data indicated that compared with the control group, there were a total of 1,329 abnormally expressed circRNAs (absolute fold change > 2) in NARDS plasma, of which 741 circRNAs were downregulated and 588 were upregulated.

### Bioinformatic Analysis of Differentially Expressed CircRNAs

**Figure 3:**
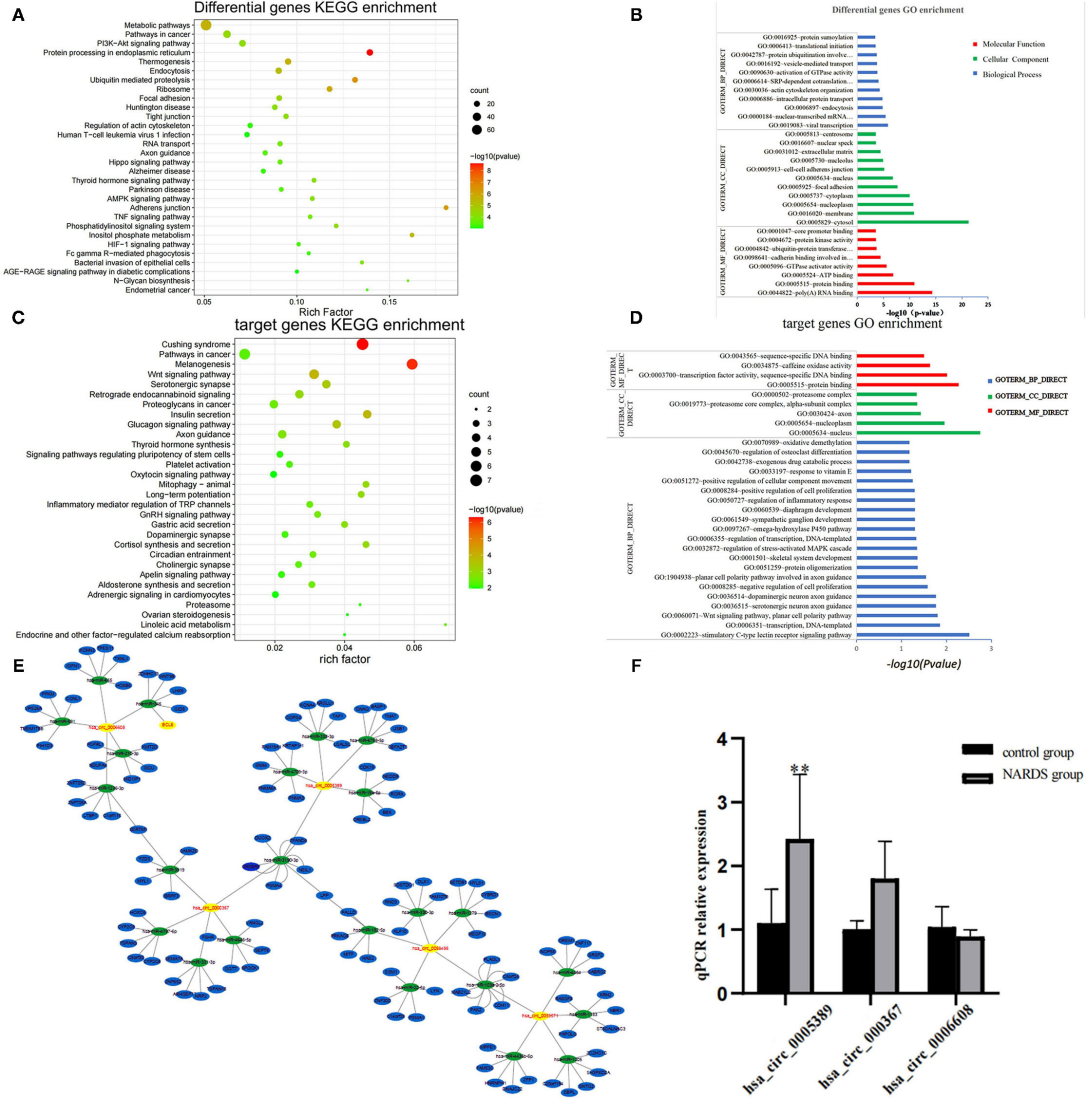
Bioinformatics analysis. **(A)** The y-axis represents the top 30 enriched KEGG pathways of differentially expressed circRNAs in NARDS. The x-axis stands for the enriched factors. **(B)** Top 30 GO-enriched terms based on the parent genes of differentially expressed circRNAs are demonstrated on the y-axis, and the negative logarithm of the *p*-value of enriched terms is shown on the x-axis. **(C)** Top 30 KEGG pathways of target genes were described according to the enrich factors of enriched target genes. **(D)** Top 30 enriched GO terms according to the negative logarithm of the *p*-value of enriched target genes. **(E)** Bioinformatics prediction of circRNA–miRNA–mRNA network. **(F)** RT-qPCR validation of selected circRNAs. It has a similar reflection to the drift of three differentially expressed circRNAs and hsa_circ_0005389 has a significant statistical difference. KEGG, Kyoto Encyclopedia of Genes and Genomes; GO, Gene Ontology; miRNA, microRNA; mRNA, messenger RNA; **p < 0.01.

The main source of circRNAs is the variable shearing of pre-mRNA, so the functions of circRNAs are associated with their parent genes (11). Therefore, parent genes of differentially expressed circRNAs were subjected to KEGG and GO analyses, which are involved in biological processes, cellular components, molecular functions, and biological pathways. The results showed the top 30 KEGG pathways (Figure 3A) and the top 30 enriched GO terms (Figure 3B), such as protein processing in the endoplasmic reticulum and ubiquitin-mediated proteolysis. It suggested that circRNAs may be related to the process of protein synthesis and metabolism in NARDS, which are potentially contributed to the inflammation of NARDS.

### Bioinformatics Prediction of circRNA–miRNA–mRNA Network

To investigate the potential molecular functions of the circRNAs, the five circRNAs including three upregulated circRNAs (hsa_circ_0005389, hsa_circ_0000367, hsa_circ_0059571) and two downregulated circRNAs (hsa_circ_0058495, hsa_circ_0006608) were selected (Table 3). Moreover, the interactions between five circRNAs and miRNAs were predicted by TargetScan and RNAhybrid, among which a total of 25 miRNAs were chosen. Later, they were used to predict target genes and 125 target genes were selected. The relationships among 5 circRNAs, 25 miRNAs, and 125 target genes were demonstrated through Cytoscape software, as shown in Figure 3E. In addition, the KEGG pathway (Figure 3C) and GO term analyses (Figure 3D) of the 125 target genes were conducted to gain insight into the five circRNAs. The top 30 enriched KEGG pathway analyses suggested that these target genes are widely involved in the synthesis and secretion of endocrine hormones, like cortisol, which is a part of the stress response. The stress response is conducive to the development of inflammation in the early stage. The top 30 enriched GO terms are focused on protein and DNA processing, like the regulation of stress-activated MAPK cascade, which has a similar reflection to the KEGG pathway analysis of the 125 target genes.

**Table 3:** Biological information of the selected five circRNAs.

**circRNA ID**	**Fold change**	**circRNA type**	**Chromosome**	**Best transcript^**#**^**	**Gene symbol**
**Upregulated**					
hsa_circ_0005389	23.9004968	Exonic	chr17	NM_001037984	SLC38A10
hsa_circ_0000367	17.5895361	Exonic	chr11	NM_001199922	SIAE
hsa_circ_0059571	14.9549406	Exonic	chr20	NM_020343	RALGAPA2
hsa_circ_0005927	13.2729292	Exonic	chr8	NM_005662	VDAC3
hsa_circ_0001666	13.258765	Exonic	chr6	NM_032448	FAM120B
**Downregulated**					
hsa_circ_0037798	−14.356974	Sense overlapping	chr16	NM_014316	CARHSP1
hsa_circ_0058495	−11.6219146	Exonic	chr2	NM_001167608	RHBDD1
hsa_circ_0006608	−10.3720939	Exonic	chr10	NM_001242339	PFKP
hsa_circ_0074306	−9.7432442	Exonic	chr5	NM_005219	DIAPH1
hsa_circ_0000981	−9.6326569	Intronic	chr2	ENST00000175091	LAPTM4A

*#The best transcript refers to the transcription of circular RNA from the same gene position; SLC38A10, solute carrier family 38 member 10; SIAE, sialic acid acetyl esterase; RALGAPA2, Ral GTPase activating protein catalytic subunit alpha 2; VDAC3, voltage-dependent anion channel 3; FAM120B, family with sequence similarity 120B; CARHSP1, calcium-regulated heat stable protein 1; RHBDD1, rhomboid domain-containing 1; PFKP, phosphofructokinase platelet; DIAPH1, diaphanous-related formin 1; LAPTM4A, lysosomal protein transmembrane 4 alpha*.

### RT-qPCR Validation of Selected circRNAs

To identify the high-throughput microarray data, three out of five circRNAs, namely, hsa_circ_0005389, hsa_circ_0000367, and hsa_circ_0006608, were selected and their expression levels were detected in the blood samples of five newborns with ARDS and five controls using RT-qPCR, for which the primer sequences of the other two circRNAs will synthesize long DNA fragments so that they are not detected by RT-qPCR. Consequently, the result of RT-qPCR (Figure 3F) has a similar reflection to the drift of three differentially expressed circRNAs.

## Discussion

NARDS is a serious life-threatening respiratory disease that often occurs in term infants and late preterm infants (15). It can be caused by a variety of factors that are consistent with those in adults and children except perinatal factors, according to Montelux criteria. Similarly, the pathological characteristics of NARDS are inflammatory cell infiltration and increased pulmonary microvascular permeability caused by many factors, which can damage lung epithelial and endothelial cells. In addition, the exudation of protein-rich fluid results in pulmonary edema and secondary lack of pulmonary surfactant, so that atelectasis occurred and exchange of carbon dioxide and oxygen failed. To prevent NARDS, strict compliance with protective lung ventilation strategies, lower concentration of inhaled oxygen, and early weaning from ventilation are needed. For severe NARDS, it is easy to develop into BPD, a chronic lung injury disease. It is worth pointing out that the development of lung morphology runs through the neonatal stage and childhood and stops at puberty (17). Severe NARDS always accompanies DAD and the formation of oxygen free radicals, which are some of the causes why lung development is slowed down or even stagnant during the neonatal period, so BPD is an adverse outcome of a newborn with NARDS.

Although the constituent ratio and mortality rate of NARDS in hospitalized newborns at the same time are low, the mortality rate of NARDS is more than 10%, so further study about NARDS is urgently needed (18). Now, existing studies have pointed out that circRNA plays a certain role in adult acute lung injury (ALI), traumatic lung injury (TLI), and lipopolysaccharide-induced acute lung injury rat model (19–21). At the same time, it has been suggested that miRNAs are involved in the pathogenesis of NARDS and play different roles in its pathological process (22). As circRNA can act as miRNA sponge, a question emerges that whether circRNA is involved in the pathogenesis of NARDS. However, there is still no study to elaborate the relationship between circRNA and NARDS.

In this article, the role of circRNA in NARDS is studied for the first time. First of all, the study included five newborns with NARDS. They reflect some risk factors, such as boys, cesarean section, and gestational diabetes mellitus, which were in line with the risk factors of NARDS, but the disadvantage was that the number of cases was too small to fully reflect the clinical information of NARDS. Secondly, in the present study, differentially expressed circRNAs were identified in NARDS for the first time, as far as we know. Their function was predicted *via* bioinformatics analysis tools. In the GO analysis, most of the top 30 GO enriched terms are focused on the protein involved in biological processes, like sumoylation, which is complemented by KEGG analysis, such as protein processing in the endoplasmic reticulum. A change (increase or decrease) of plasma protein concentration is one of the primary characteristics of inflammation, especially in the early stage or acute phase (23). One study showed a cytokine profile in serum or bronchoalveolar lavage fluid of ARDS, in which increased acute phase markers (such as C-reactive protein) and inflammatory cytokines (for instance, TNF-a) have a consistent profile (24). What is more, the formation of protein-rich edema which is attributed to the disruption of the alveolar–capillary membrane in the air spaces is one of the main factors that result in the severe impairment of blood and tissue oxygenation early in the evolution of DAD caused by NARDS (25). Thus, circRNA is related to the pathological process of NARDS.

For further study, the five selected circRNAs were used to predict correlative miRNAs and their target genes. Then, the GO and KEGG analyses of the target genes demonstrated that they are involved in endocrine hormone and inflammation, in which the result of the top 30 enriched GO terms is centered on protein and DNA processing. Glucocorticoids (GCs) occupied the most places in the top 30 KEGG pathways, in which a variety of stress-related hormones have been mentioned to regulate the expression of various genes and miRNAs, and it is not only an anti-inflammation ingredient but also a pro-inflammation component (26, 27). Although the balance between pro-inflammatory mediators and anti-inflammatory mediators determines the inflammatory response, stress conditions increase the migration and the survival of neutrophils by GCs (24, 28), which could promote the development of inflammation in the early stage of NARDS (27). In addition, previous studies indicate that GCs can induce an inflammatory response which induces a pro-inflammatory shift in the balance of IL-1β and anti-inflammatory secreted IL-1 receptor antagonist (sIL-1Ra) in neutrophils (29), so the target genes of correlative miRNA of the five selected circRNAs may contribute to the inflammation in the early stage of NARDS. In addition, an RT-qPCR validation of three circRNAs was carried out in the 10 blood samples and hsa_circ_0005389 has a significant statistical difference (*p* < 0.01), which is a similar result to microarray. Interestingly, its gene symbol is solute carrier family 38 member 10 (SLC38A10), which is also related to the protein process. SLC38A10 is an amino acid transporter and plays a role in regulating nascent protein synthesis and cell survival under oxidative stress (30). Thus, hsa_circ_0005389 will be used for deeper study in future articles.

Taken together, the study profiles differentially expressed circRNAs in newborns with ARDS. Our finding showed for the first time that differentially expressed circRNAs participate in the pathogenesis of NARDS, which may provide a novel potential therapeutic direction and a new idea for early diagnosis of NARDS showing that circRNAs can be used as molecular markers. The current study has some limitations: the specific mechanism of circRNAs in NARDS has not been studied and the sample size of the RT-qPCR and microarray is small. Besides, the result of RT-qPCR has a higher bar because of individual heterogeneity. In the near future, more work is needed to explore the specific role of differentially expressed circRNAs in NARDS.

## Data Availability Statement

The raw data supporting the conclusions of this article will be made available by the authors, without undue reservation.

## Ethics Statement

The studies involving human participants were reviewed and approved by the Clinical Research Ethics Committee of the First Affiliated Hospital of Nanjing Medical University. Written informed consent to participate in this study was provided by the participants' legal guardian/next of kin.

## Author Contributions

YY and X-qC designed the study. HZ wrote the primal draft of the manuscript, which was revised by X-qC, YY, and RC. HZ, X-jW, M-yY, and Y-hZ collected and analyzed the data. HZ, BC, and Y-fC contributed to all figures and tables. All authors read and approved the final manuscript.

## Funding

This work was funded by the National Natural Science Foundation of China (Nos. 81871195 and 81741052) and the Health Research Project of Maternal and Child Health of Jiangsu Province Hospital (F201745).

## Conflict of Interest

The authors declare that the research was conducted in the absence of any commercial or financial relationships that could be construed as a potential conflict of interest.

## Publisher's Note

All claims expressed in this article are solely those of the authors and do not necessarily represent those of their affiliated organizations, or those of the publisher, the editors and the reviewers. Any product that may be evaluated in this article, or claim that may be made by its manufacturer, is not guaranteed or endorsed by the publisher.
